# Development and validation of a simple risk model to predict major cancers for patients with nonalcoholic fatty liver disease

**DOI:** 10.1002/cam4.2777

**Published:** 2019-12-20

**Authors:** Zihan Wei, Zhigang Ren, Shuang Hu, Yan Gao, Ranran Sun, Shuai Lv, Guojie Yang, Zujiang Yu, Quancheng Kan

**Affiliations:** ^1^ Department of Geriatrics The First Affiliated Hospital of Zhengzhou University Zhengzhou China; ^2^ Department of Infectious Diseases The First Affiliated Hospital of Zhengzhou University Zhengzhou China; ^3^ Gene Hospital of Henan Province Precision Medicine Center The First Affiliated Hospital of Zhengzhou University Zhengzhou China; ^4^ Department of Pharmacy The First Affiliated Hospital of Zhengzhou University Zhengzhou China; ^5^ National Clinical Research Center of Cardiovascular Diseases State Key Laboratory of Cardiovascular Disease Fuwai Hospital National Center for Cardiovascular Diseases Beijing China; ^6^ Department of gastroenterology The First Affiliated Hospital of Zhengzhou University Zhengzhou China

**Keywords:** cancers, hepatocellular carcinoma, nonalcoholic fatty liver disease, risk model

## Abstract

**Objective:**

To recognize risk factors and build up and validate a simple risk model predicting 8‐year cancer events after nonalcoholic fatty liver disease (NAFLD).

**Methods:**

This was a retrospective cohort study. Patients with NAFLD (n = 5561) were randomly divided into groups: training (n = 1254), test (n = 627), evaluation (n = 627), and validation (n = 3053). Risk factors were recognized by statistical method named as a Cox model with Markov chain Monte Carlo (MCMC) simulation. This prediction score was established based on the training group and was further validated based on the testing and evaluation group from January 1, 2007 to December 31, 2009 and another 3053 independent cases from January 1, 2010 to February 13, 2014.

**Results:**

The main outcomes were NAFLD‐related cancer events, including those of the liver, breast, esophagus, stomach, pancreas, prostate and colon, within 8 years after hospitalization for NAFLD diagnosis. Seven risk factors (age (every 5 years),LDL, smoking, BMI, diabetes, OSAS, and aspartate aminotransferase (every 5 units)) were identified as independent indicators of cancer events. This risk model contained a predictive range of 0.4%‐37.7%, 0.3%‐39.6%, and 0.4%‐39.3% in the training, test, evaluation group, respectively, with a range 0.4%‐30.4% for validation groups. In the training group, 12.6%, 76.9%, and 10.5% of patients, which corresponded to the low ‐, moderate ‐, and high‐risk groups, had probabilities of, <0.01, <0.1, and 0.23 for 8‐year events.

**Conclusions:**

Seven risk factors were recognized and a simple risk model were developed and validated to predict the risk of cancer events after NAFLD based on 8 years. This simple risk score system may recognize high‐risk patients and reduce cancer incidence.

## Graphical abstract

Seven risk factors including age (every 5 years), low‐density lipoprotein‐cholesterol, smoking, body mass index, diabetes, obstructive sleep apnea syndrome, and aspartate aminotransferase (every 5 units) were independent indicators of 8‐year cancer events in patients with NAFLD. A simple risk model and a score system were developed to predict the risk of cancer events after NAFLD based on 8‐year follow‐up of 1254 NAFLD patients. The risk model was well validated in the test samples (n = 627) and evaluation samples (n = 627) with 8‐year follow‐up. Importantly, the risk model performed well in another independent validation samples (n = 3053). This risk model provides a basis for clinicians to better understand patients' risk of long‐term cancer events after NAFLD.

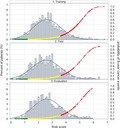

**Abbreviations**
BMI: body mass index
HDL: High‐density lipoprotein
LDL: Low‐density lipoprotein
MCMC: Markov chain monte carlo
MS: metabolic syndrome
NAFL: Nonalcoholic fatty liver
NAFLD: Nonalcoholic fatty liver disease
NASH: nonalcoholic steatohepatitis
OSAS: obstructive sleep apnea syndrome
RDI: respiratory disturbance index
ROC curves: receiver operating characteristic curves
ROS: reactive oxygen species

## 1 INTRODUCTION

Nonalcoholic fatty liver disease (NAFLD) has a high prevalence and increasing morbidity in China and other countries.^1^, ^2^, ^3^, ^4^ Patients with NAFLD are at high risk of developing cancers, such as primarily hepatocellular carcinoma, colorectal cancer, or breast cancer.^5^, ^6^ NAFLD‐related cancers have been causing a significant burden for healthcare in China and other countries.^7^, ^8^ It has been shown that patients suffering from NAFLD show high potential for cardiovascular diseases and cancer development, which remains the main cause of death among patients with NAFLD.^9^ However, few patients are aware of the severe outcomes of NAFLD, partly because of its common benign course with a low risk of aggravating cirrhosis. Thus, different prognoses in different risk stratifications and histopathological subtypes are frequently ignored.^10^

Although the risk factors for NAFLD‐relevant hepatocellular carcinoma 11 and a noninvasive model predicting liver fibrosis have been determined and investigated in the West,^12^, ^13^ the establishment of a specific model to forecast NAFLD‐associated cancer is still urgently needed, especially based on the elderly Chinese cohort. One explanation is that risk factors (such as age, sex, and body mass index) for cancers are not identical between China and developed countries. In addition, NAFLD‐relevant carcinomas were ubiquitously ignored, compared with the prediction of liver fibrosis in previous studies. In the majority of patients, NAFLD is bidirectionally associated with metabolic risk factors (such as central obesity, diabetes mellitus, dyslipidemia, and hypertension), which might represent an important etiology of the increasing morbidity of various solid tumors beyond that of liver cancer.^14^, ^15^ Furthermore, a number of studies focus predominately on the mortality of NAFLD with a long‐term history.^16^ In contrast, a focus on the onset and prophylaxis of various cancers, rather than an investigation of the mortality of cancer, will provide much more significant clinical outcomes.^5^, ^16^ Therefore, much more importance should be attached to the long‐term outcomes than to the mortality of NAFLD to determine the complete healthcare experience of patients, particularly in developing countries. The determination of cancerous features helps patients and clinicians predict the future risk of cancer, thus promoting intensive follow‐up and risk factor adjustment as well as further relief of the financial pressures caused by the high incidence of cancer.

Accordingly, our study developed and evaluated a simple risk model by identifying significant clinical risk factors to predict NAFLD‐related cancers on the basis of 5561 patients with NAFLD in the First Affiliated Hospital of Zhengzhou University, the largest tertiary medical hospital in China, which has approximately 10 000 beds, 15 000 inpatients/ day and 20 000 outpatients/ day. Patients with NAFLD diagnosed between 1/1/2007 and 2/13/2014 were included and followed until cancer diagnosis, death, or through 12/31/2017. In this cohort, all the samples were extracted from medical records about patients with NAFLD and were well designed to further stratify risk after NAFLD diagnosis. The main endpoint of the study was to predict the presence or absence of cancers by a combination of simple and clinically relevant variables in the elderly Chinese population.

## 2 METHODS

### 2.1 Study Group

A total of 5561 patients with NAFLD in this study were first confirmed by ultrasound. The radiological features of these patients included fatty liver and increased or heterogeneous echogenicity. They visited the First Affiliated Hospital of Zhengzhou University between January 1, 2007 and February 13, 2014 more than two times and were followed until cancer diagnosis, death or through December 31, 2017. Medical records were extracted individually by three doctors; consistency was 97% for main data elements. Based on 8‐year follow‐up of NAFLD patients, information on all types of cancer events was collected from medical records, including liver, breast, esophagus, stomach, pancreas, prostate, and colon cancers.

Patients with liver disease of other etiologies were appropriately excluded, including autoimmune or viral hepatitis, alcohol‐induced, or drug‐induced liver disease and cholestatic or genetic liver disease. These other liver diseases were excluded applying specific clinical, biochemical, radiographic, and/or histological criteria. All patients had a negative history of ethanol abuse, as indicated by a weekly ethanol consumption of ≤140 g in women and ≤210 g in men. A history of alcohol consumption was specifically investigated from medical records. Patients with clinical or imaging evidence of decompensated cirrhosis were specifically excluded from this study because they most likely had cirrhotic‐stage NAFLD.

**Figure 1:**
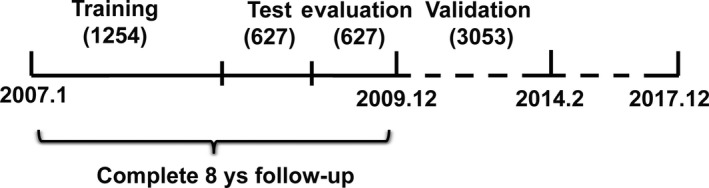
The design of the study. The group from 2007 to 2009 included 2508 unique patients who we randomly divided into three independent groups for training (50% [1254 patients]), test (25% [627 patients]), and evaluation (25% [627 patients]) analyses. The training group was used to select risk factors; the test and evaluation group were used for subsequent evaluation. The other independent group from 2010 to 2014 included 3053 unique patients for further validation analysis

The cohort from 2007 to 2009 included 2508 patients. These samples were randomly divided into three independent groups named as training group(50% [1254patients]), test group(25% [627patients]), and evaluation group(25% [627 patients]). The training group was used to identify risk factors of cancer events for NAFLD patients with 8‐year follow‐up. The test group and evaluation group were used for validation. The other independent cohort from 2010 to 2014 enrolled a total of 3053 unique patients for further validation analysis. This study was agreed by the Institutional Review Board of the First Affiliated Hospital of Zhengzhou University (Figure 1).

### 2.2 Potential risk factors

The candidate risk factors contained clinical and laboratory data were easily and dependable collected within hospitalization for NAFLD as well as were selected by their clinical meaning, supported document. Not only detailed medical history but also entire physical examination were abstracted from patients. Initial factors included patient demographics (age, sex, and body mass index calculated with the formula weight (in kilograms)/height (in meters^2^)), medical history (hypertension, diabetes mellitus, obstructive sleep apnea syndrome, family history of cancer, hyperlipoidemia), lifestyle factors (smoking, drinking), laboratory evaluation including routine liver biochemistry (alanine aminotransferase and aspartate aminotransferase levels, total bilirubin, albumin, and alkaline phosphatase), complete blood count, total cholesterol, HDL cholesterol, LDL cholesterol, and total triglycerides.

The definitions of comorbidity that were used in this study included the following: hypertension (systolic blood pressure ≥140, diastolic blood pressure ≥90 or treatment of previously diagnosed hypertension); diabetes mellitus (fasting glucose ≥126 mg/dL or treatment with antidiabetic drugs); and obstructive sleep apnea syndrome (based on the respiratory disturbance index (RDI) ≥5 obstructive events/h of sleep, for which patients were diagnosed with obstructive sleep apnea syndrome).

### 2.3 Outcome

In this risk model, the outcome was 8‐year cancer events, a binary variable recognized as the occurrence of cancers, including those of the liver, breast, esophagus, stomach, pancreas, prostate, and colon, within 8 years of diagnosis for NAFLD. Messages on the outcome were received and acknowledged by medical records.

## 3 STATISTICAL ANALYSIS

### 3.1 Risk factor selection and test

In the training group, we fitted the statistical MCMC simulation and computed a posterior probability for the whole risk factors.^17^ The posterior probability judges the strength of a correlation between a factor and the outcome. A factor with a posterior probability more than 0.95 was regarded statistical significant for predicting 8‐year cancer events and contained in the ultimate risk factor register.^18^ We developed the ultimate risk model to predict outcome by matching the Cox model to the training group, using the selected risk factors. Routine demographic, comorbidity, and laboratory variables were analyzed by multivariate modeling to predict the presence or absence of 8‐year cancer events.

We test this predictive model performance by the following statistics method: Harrell's c‐statistic to evaluate the total accuracy of prediction,^19^ ROC curves depended on time to evaluate the predictive accuracy during 8 years,^20^ partial residuals and Hosmer and Lemeshow's Goodness of Fit Test Statistic to evaluate the proportional hazards assumption and calibration,^21^ and Schemper and Henderson measurement to estimate explained variation.^22^ Distinction was evaluated within the observed cancer events by stratification described as deciles of predictive probabilities.^23^ In the training group, we divided samples into 10 independent risk grades on the basis of these deciles, classifying the grades from minimum risk to maximum risk for validation.^24^

Additionally, this predictive model performance was evaluated and compared in the test, evaluation, and validation group, respectively.

### 3.2 Risk score

For the convenient application of elected risk factors and this predictive model, we developed a easily applied score system for every patient with NAFLD on the basis of the regression coefficients assessed from the predictive model with the training group. The scores for every risk factor were counted by grading the coefficient of risk factor by the sum of all coefficients in this model, multiplying by 100, meanwhile rounding to the nearest integer. Then, the risk score was calculated by summing points of patients.^24^, ^25^, ^26^ Furthermore, we classed patients with NAFLD into three risk groups of cancer events based on the spread of this score: high‐risk group(>90th percentile), moderate‐risk group (10th‐90th percentile), and low‐risk group (<10th percentile). Analyses were conducted between August 10, 2018 and November 22, 2018 using SAS version 9.4.

## 4 RESULTS

### 4.1 Study cohort

A total of 5561 (1254 training, 627 test, 627 evaluation and 3053 validation) patients were enrolled. The mean age was 69.4 years (standard deviation [SD] 8.1), and 49.4% were female. The common comorbidities were diabetes mellitus (27.2%), obstructive sleep apnea syndrome (19.2%), hypertension (27.8%), and dyslipidemia (70.5%). There were not significant differences in basic characteristics of patients across the training, test, and evaluation group. However, the common comorbidities in validation groups were higher than in the other three groups (Table 1).

**Table 1:** Patient characteristics by training, test, evaluation, and validation groups

Characteristics	2007‐2009 (development, n = 2508)			2010‐2014 (Validation, n = 3053)
	Training	Test	Evaluation	Validation
Number of patients	1254	627	627	3053
Demographics				
Female, # (%)	633 (50.5)	317 (50.6)	301 (48.0)	1496 (49.0)
Age ≥ 65 years, # (%)	906 (72.3)	457 (72.9)	442 (70.5)	2248 (73.6)
Mean age (SD)	69.2 (8.1)	69.5 (8.0)	69.2 (8.2)	69.6 (8.1)
Medical history or comorbidity, # (%)				
Diabetes mellitus # (%)	273 (21.8)	138 (22.0)	141 (22.5)	962 (31.5)
Obstructive sleep apnea	212 (16.9)	82 (13.1)	96(15.3)	676 (22.1)
Syndrome, # (%)				
Smoking, # (%)	188 (15.0)	96 (15.3)	104 (16.6)	581 (19.0)
Family history of cancer # (%)	54 (4.3)	21 (3.4)	49 (7.8)	156 (5.1)
Hypertension # (%)	289 (23.1)	162 (25.8)	162 (25.8)	932 (30.5)

### 4.2 8‐Year cancer events

**Figure 2:**
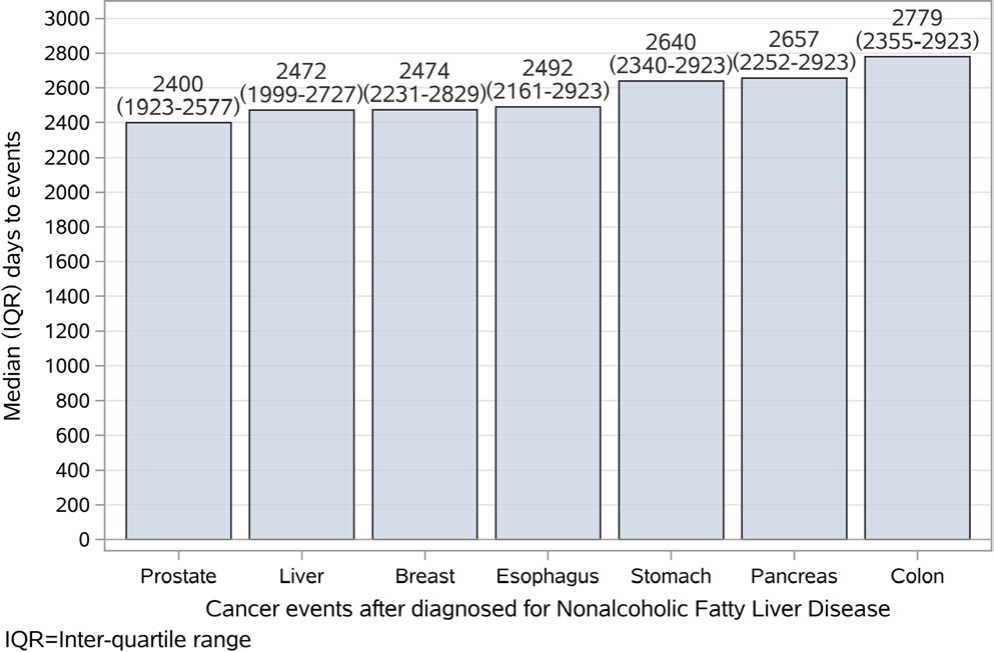
The median (IQR) days from diagnosed for NAFLD to a cancer event was 2533 (2179‐2923) days. Prostate and liver occurred earlier than other primary areas of cancer (median 2400 [1923‐2577] days for prostate and median 2472 [ 1999‐2727]) days for liver

The rates of 8‐year cancer events were 10.1% (95% [CI] 8.5‐11.9), 10.2% (95% [CI] 7.9‐12.8), and 10.2% (95% [CI] 7.9‐12.8) for the training, test, and evaluation group, respectively. Overall, the nonfatal cancer event rate was 10.2%. The median [interquartile range (IQR)] number of days from NAFLD diagnosis to a cancer event was 2533 (2179‐2923) days; prostate and liver occurred earlier than other primary areas of cancer (median 2400 [1923‐2577] days for prostate cancer and median 2472 [1999‐2727] days for liver cancer; Figure 2).

### 4.3 Risk factors selection and evaluation

The MCMC simulation identified seven candidate factors that had a posterior probability <.95 (Table 2), including age (every 5 years), body mass index, diabetes mellitus, obstructive sleep apnea syndrome, smoking, LDL, and aspartate aminotransferase (every 5 units) (Figure S1). According to these seven risk factors, the risk model was developed and the training group showed good differentiation and calibration. The total c‐statistic of this predictive model was 0.94. The average observed 8‐year outcome of predicted decile extended from 0.4% to 37.7% (Figure S2). The Hosmer and Lemeshow's Goodness of Fit Test’ *p* value in the training group was .77, in the test group was .78, and in the evaluation group was .98 meaning that the predicted cohort was well matched with the observed cohort (Figure S3). Schemper and Henderson measurement was 0.51 as well as partial residuals test represented that each of risk factors satisfied the proportional hazards assumption.

**Table 2:** Final risk prediction model for 8‐year cancer event after diagnosed for NAFLD based on training group

Risk factor	Training data		
	Regression coefficient	Hazard ratio (95% CI)	Points*
Age_5	0.313	1.37 (1.19‐1.58)	9
BMI	0.277	1.32 (1.15‐1.51)	8
DM	0.840	2.32 (1.40‐3.82)	23
OSAS	0.623	1.86 (1.15‐3.02)	17
AST_5	0.225	1.25 (1.17‐1.34)	6
Smoking	0.418	1.52 (0.96‐2.39)	12
LDL	0.890	2.44 (1.67‐3.56)	25

Note

Age_5: Age (every 5 years).

Abbreviations: BMI, body mass index; DM, diabetes mellitus; OSAS, obstructive sleep apnea syndrome; AST_5, aspartate aminotransferase ( every 5 units); LDL, low‐density lipoprotein.

* Points were calculated by dividing a risk factor's coefficient by the sum of all coefficients, multiplying by 100, and rounding to the nearest integer.

The model also performed well in the test and evaluation group in accordance with the training group. The total c‐statistic was 0.91 and 0.92 in the test and evaluation group, respectively; the rate of cancer events following up 8 years in the observed samples extended from 0.03% to 39.6% and the rate of that in the predicted samples from 0.04% to 39.3%.

### 4.4 Risk score system

**Figure 3:**
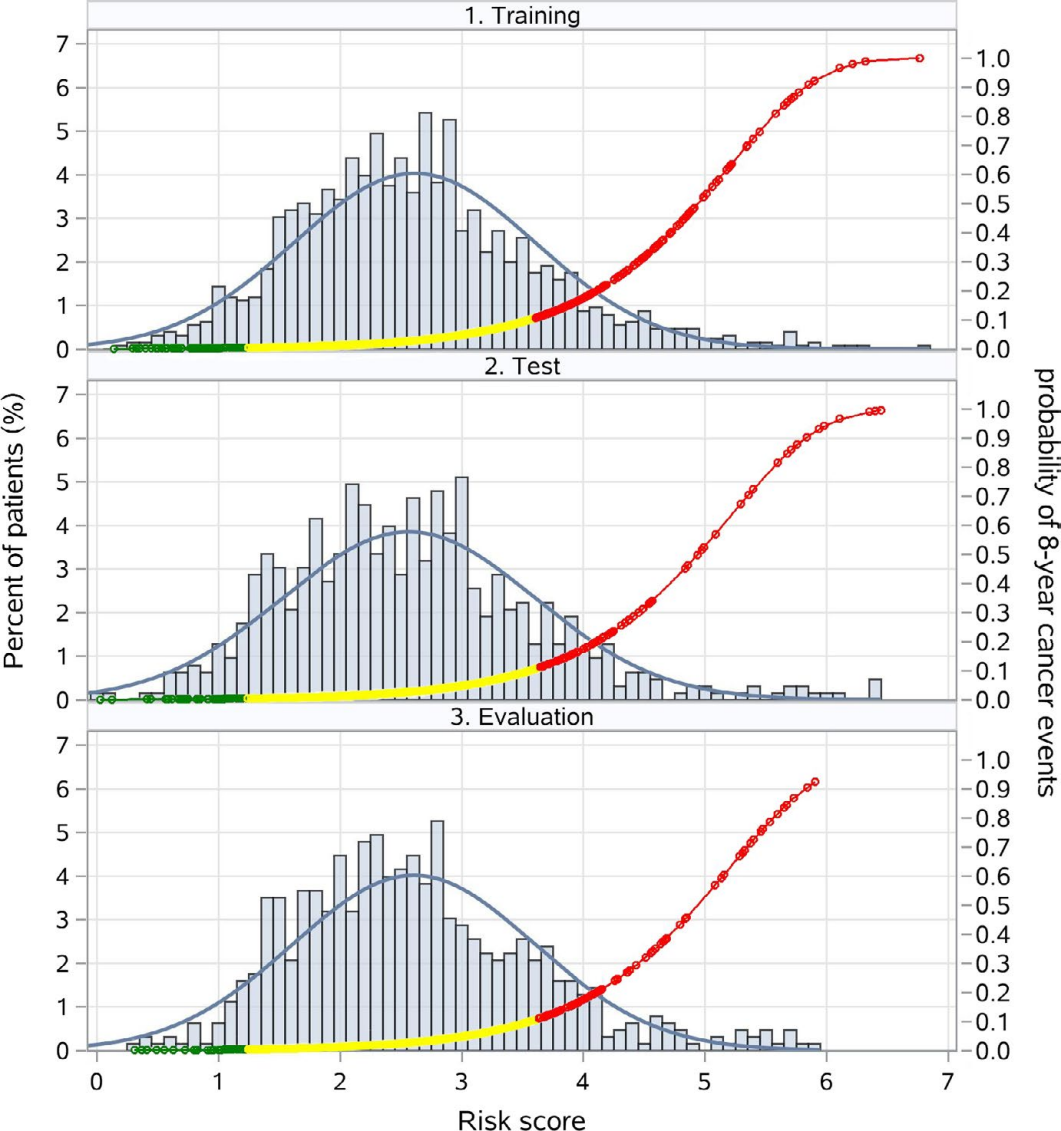
Distribution of patient risk scores in the training, test, and evaluation group (histograms) and probability of 8‐year cancer event by risk score (curves). The yellow curve represents a fitted density curve on the risk score histogram. A risk score, ranging from 0 to 100, was constructed at the patient level based on the regression coefficients estimated from the final risk model with the training group. A higher risk score indicates a higher probability of developing 8‐year after diagnosed for cancer event

**Figure 4:**
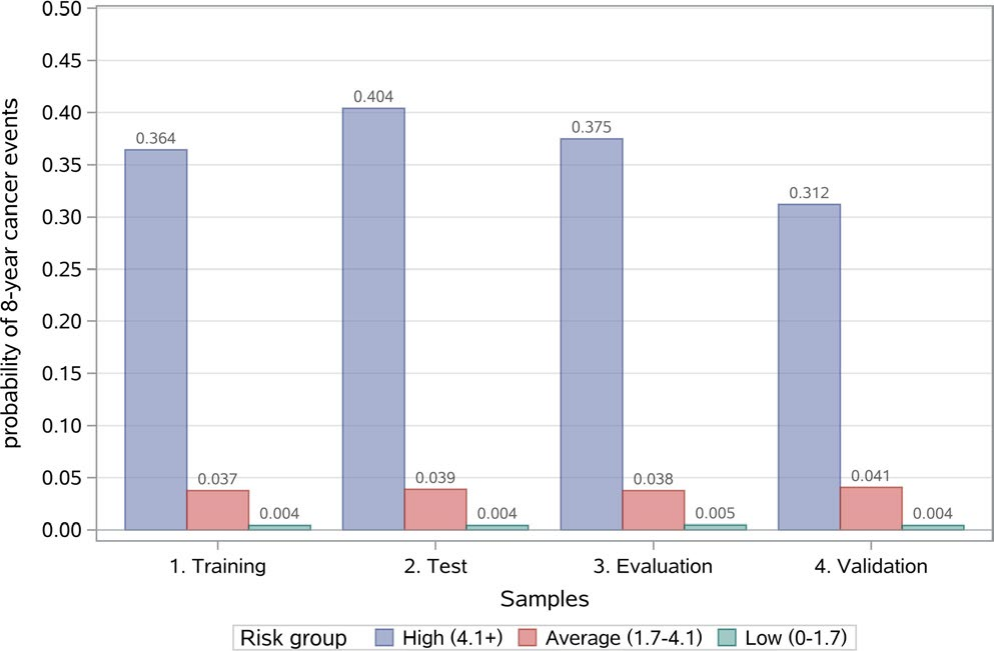
Risk stratification by risk scores. For the training, test, evaluation and validation groups, respectively, the highest risk group includes 10.5%, 9.6%, and 10.7% and 9.5% of the patients, the moderate risk group includes 76.9%, 73.8%, 75.4% and 79.6% of the patients, and the lowest risk group includes 12.6%, 16.6%, and 13.9% and 10.8% of the patients

The points of risk factors extended from 6 (aspartate aminotransferase every 5 units) to 25 (LDL) (Table 2). The training group had a average risk score of 2.85 (SD 0.99). The average score was 2.80 (SD 1.03) for the test group and 2.85 (SD 0.99) for the evaluation group (Figure 2). In the training group, 12.6%, 76.9%, and 10.5% of patients were stratified into the low‐, moderate‐, and high‐risk groups, respectively, in accordance with probabilities of < 0.01, <0.1, and 0.23 for outcomes of 8 years (Figure 3). The stratifications for the test and evaluation group were similar to those for the training group (Figures 3 and 4; Table S1).

### 4.5 Validation

For the validation group, the rates of cancer events were 2.2% (95% confidence interval [CI] 1.7‐2.8). The average observed cancer outcome extended from 0.4% to 30.4% in the predicted decile (Figure S2). The Hosmer and Lemeshow's Goodness of Fit Test’ *p* value was 0.21 (Figure S3).

For the validation group, the mean (SD) of the risk score was 2.90 (SD 0.91) (Figure S4). In the validation group, 10.8%, 79.6%, and 9.5% were stratified into the low‐, moderate‐, and high‐risk groups, respectively, in accordance with probabilities of <.01, <.1, .31 and for cancer events (Figure 3). The probability of 8‐year cancer events in high‐risk group in the validation group was lower than those for the training group, whereas the probability of 8‐year cancer events in moderate‐ and low‐risk groups were similar to those for the training group (Figure 4 and Figure S4; Table S1).

## 5 DISCUSSION

In this large cohort study, we found that seven risk factors, including age (every 5 years), low‐density lipoprotein‐cholesterol, smoking, body mass index, diabetes, obstructive sleep apnea syndrome, and aspartate aminotransferase (every 5 units), were independent indicators of 8‐year cancer events in patients with NAFLD. This simple risk model and the score system were developed and validated to predict 8‐year cancers NAFLD diagnosis. Importantly, the risk model performed well in another independent validation NAFLD patients. These factors were selected on the basis of data selected from medical records and ease of collection and ready availability at the time of discharge as well as long‐term follow‐up. Furthermore, statistical algorithms in this study is robust. Not only this predictive model but also this score system help clinicians recognize patients with NAFLD at increased risk of 8‐year cancers and assist patients understand their risk of cancers. The capability to recognize patients with the highest risk of cancers after NAFLD diagnosis may provide targeted, higher‐quality, and intensive healthcare after discharge.

Our study, based on information selected from medical records and continuing for 8 years of follow‐up, presents a large cohort study of risk factors that predict the spread of outcomes for senior citizens with NAFLD in the central plains of China. Furthermore, the patients represented in these data always visit the same hospital many times to acquire the comprehensive and professional treatment and healthcare in this general teaching and urban hospital. Importantly, evidence shows that most of the patients developing NAFLD present at least one of the traits of metabolic syndrome (MS).^27^, ^28^ Several studies indicate a potent association between metabolism syndrome and the risk of certain types of cancer, in addition to hepatocellular carcinoma.^29^ However, previous studies and different types of risk scores examining advanced liver fibrosis or the natural history of NAFLD originate from specialist centers in which patients had been mostly selected from developed countries.^30^, ^31^, ^32^, ^33^ In this study, we validated and classified the risk of NAFLD‐associated different types of cancers, comparing with prior studies which only focused on advanced fibrosis.

The MCMC algorithm was used to evaluate the strength of the association between the risk factors and the outcome. On the basis of data from 2007 to 2009, we developed and evaluated this simple noninvasive predictive risk model. In contrast to other studies, our study had better predictive accuracy based on another independent cohort of patients with NAFLD from January 1, 2010 to February 13, 2014, which was used to revalidate this scoring system.^12^, ^33^ NAFLD may evolve into a tumor, but it is easily overlooked in the stage of fatty liver. Our predictive model was constructed on the characteristics and comorbidities at baseline of patients with NAFLD, and it is simple to use. The previous studies that constructed predictive scores with some biomarkers were inconvenient to review periodically.^33^, ^34^ The lack of availability of these serum markers of fibrosis in most centers makes it difficult to apply the proposed scoring system on a daily basis.^35^, ^36^

Our risk factors were recognized on the basis of a large cohort that always visited and followed up in the same hospital for many times. Data for an effective risk factor ought to be stabilized by clinical illustration, conveniently collected, widely received during hospitalization and at discharge. In this study, these seven risk factors found fitted the whole criteria, meanwhile the majority of them have been recognized in many studies.^37^ Most of these risk factors in our study are related to metabolic dysregulation and could be improved by effective long‐term follow‐up. LDL and diabetes were the top two factors. It has been demonstrated that hyper‐LDL cholesterol is associated with colorectal adenomas, breast cancer, and prostate and liver cancer.^38^, ^39^ It is said that persons with diabetes, rather than only obese individuals, are apt to develop cancers.^40^ In 2010, there was convincing evidence that diabetes, either alone or as a cofactor, was associated with an increased risk of liver, colorectal, pancreatic, and breast cancer from the American Diabetes Association and the American Cancer Society.^41^, ^42^ Although NAFL steatosis is generally a benign disorder, patients with the disorder may still suffer from cancer in the presence of risk factors as determined in our study. These risk factors elevated the levels of reactive oxygen species (ROS), overloaded mitochondrial capacity for oxidative stress, and promoted DNA damage to liver tissues and other increased visceral adipose tissues by the proinflammatory signaling pathway.^43^, ^44^ In our study, many risk factors of cancers were changeable and led to different outcomes and prognoses in the long term. At baseline, patients in the high‐risk group were small, whereas patients in the moderate risk group may fall to cancer events in the long term. Given their knowledge of the risk score, patients in different stratifications should all be aware of the risks for poor prognosis as well as avoid and improve their risk factors to transition themselves from the high‐risk group to the low‐risk group. With the improvement in patients’ postdischarge outcomes and the reduction in cancer rates, the economic burden on healthcare can be relieved, and more individuals with cancer events can be saved.

All cases with NAFLD were diagnosed by abdominal ultrasound of hepatic steatosis, which provides less accuracy than diagnosis by liver biopsy. However, ultrasonographic detection has been widely used in other studies to verify fatty liver.^45^, ^46^ Additionally, only 30 percent of the 5561 patients were reported to have severity typing descriptions in ultrasonographic detection. Therefore, the predictive model and risk factors recognized in our study still need to be validated and updated.

In conclusion, this simple risk model had a robust predictive scope and could provide a basis for clinicians to better understand patients' risk of long‐term cancer events after NAFLD. It assists clinicians make better‐targeted, evidence‐based decisions for postdischarge NAFLD management.

## CONFLICTS OF INTEREST

All authors declare no conflict of interest.

## AUTHOR CONTRIBUTIONS

QK, ZY, and GY designed the study. ZW, ZR, and RS collected data; GY and SH analyzed data; ZW wrote the manuscript; ZR revised the manuscript. All authors reviewed and approved the manuscript.

## ETHICS APPROVAL AND CONSENT TO PARTICIPATE

This study was approved by the First Affiliated Hospital of Zhengzhou University (2017‐XY‐002). The study was performed in accordance with the Helsinki Declaration and Rules of Good Clinical Practice. All participants signed written informed consents after the study protocol was fully explained.

## CONSENT FOR PUBLICATION

No individual data are reported in this article.
