# Supplementary figures and images for: Development and validation of a simple risk model to predict major cancers for patients with nonalcoholic fatty liver disease
Source: Cancer Med. 2019 Dec 20;9(3):1254–62. doi: 10.1002/cam4.2777 (PMC6997093; doi:10.1002/cam4.2777)

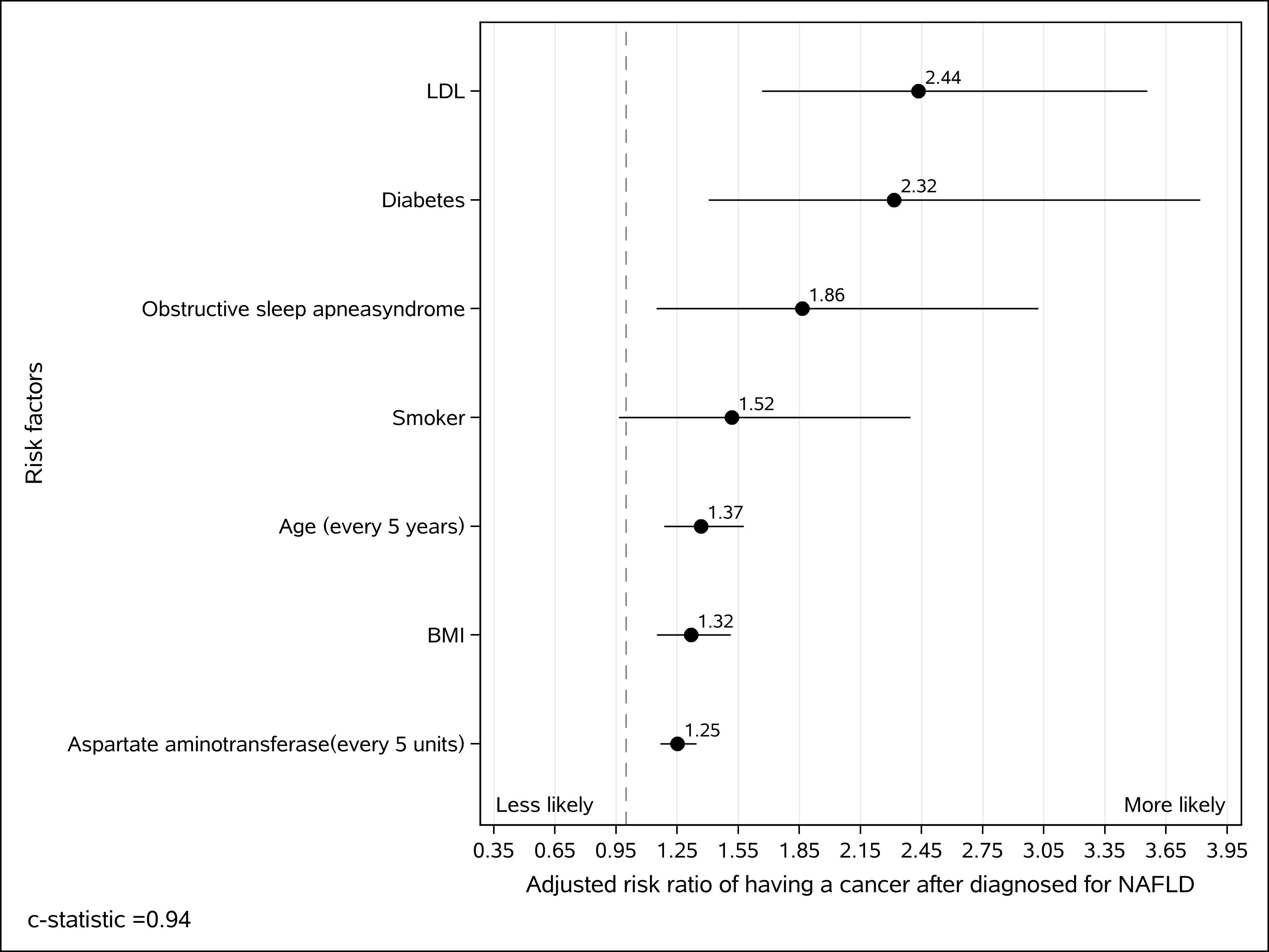

Supplement: Supplementary file 1 [file CAM4-9-1254-s001.jpeg]

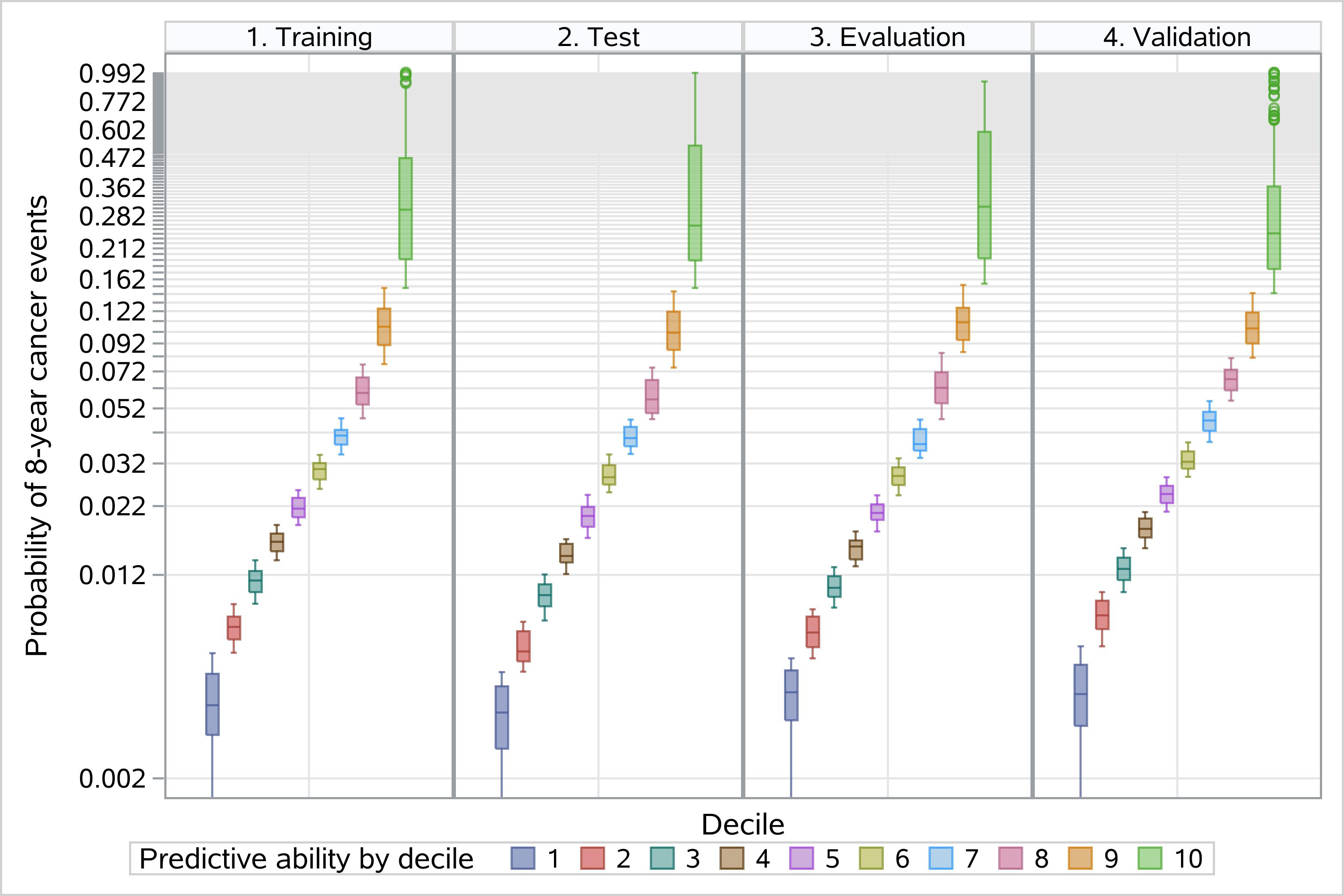

Supplement: Supplementary file 2 [file CAM4-9-1254-s002.jpeg]

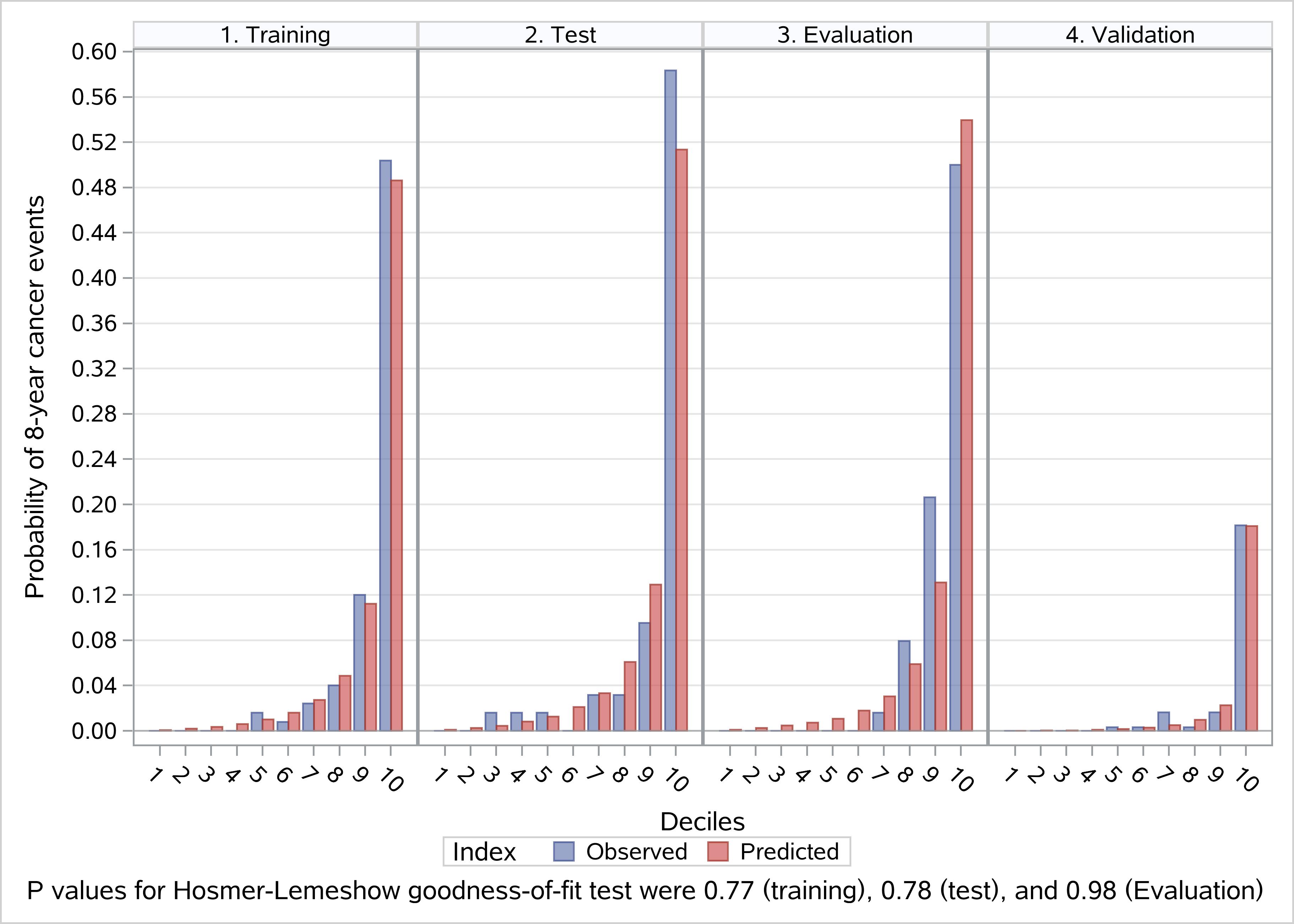

Supplement: Supplementary file 3 [file CAM4-9-1254-s003.jpeg]

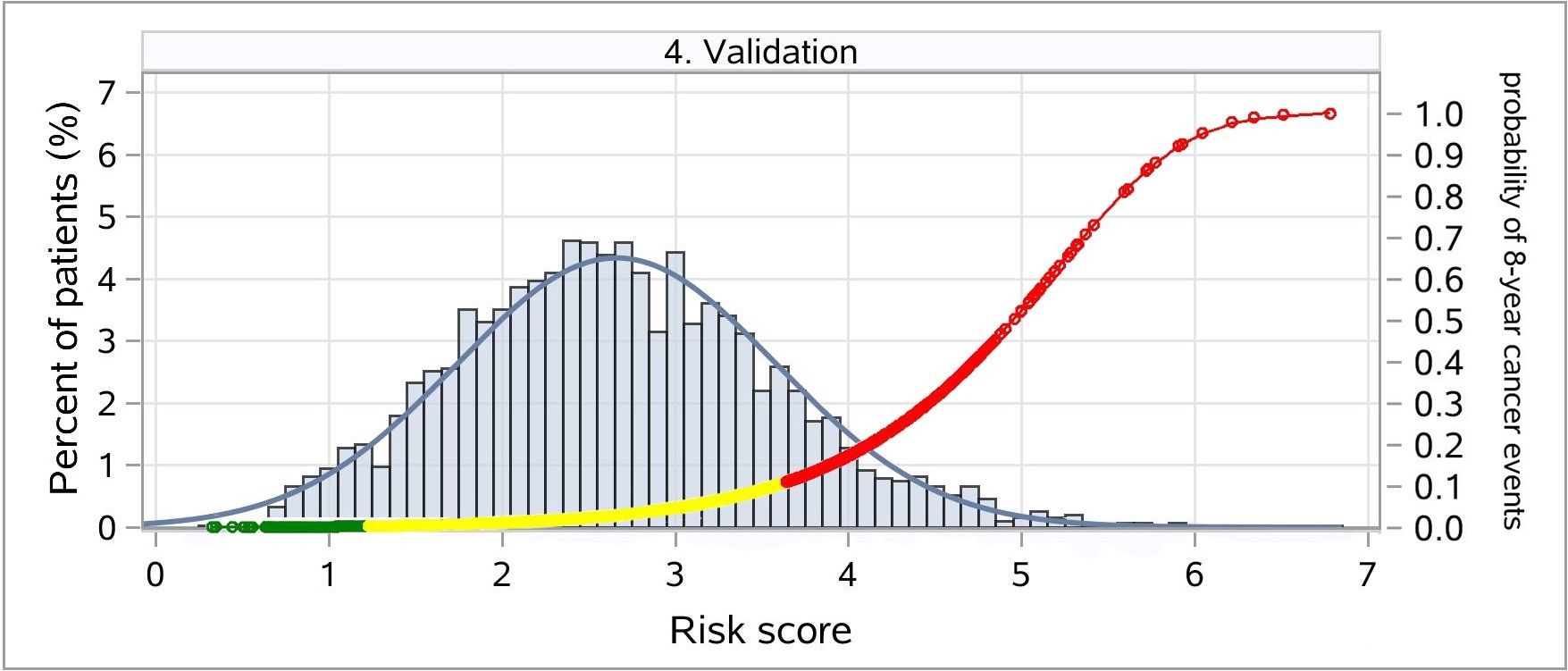

Supplement: Supplementary file 4 [file CAM4-9-1254-s004.jpg]
