# Supplementary material for: Development and validation of a simple risk model to predict major cancers for patients with nonalcoholic fatty liver disease
Source: Cancer Med. 2019 Dec 20;9(3):1254–62. doi: 10.1002/cam4.2777 (PMC6997093; doi:10.1002/cam4.2777)
Supplement: Supplementary file 5 [file CAM4-9-1254-s005.docx]

**Supplementary Table**

**eTable 1. Patient risk stratification based on risk score**

| Risk group | Training（1254） | | Test（627） | | Evaluation（627） | | Validation（3053） | |
| --- | --- | --- | --- | --- | --- | --- | --- | --- |
|  | Patients,  #(%) | Probability of 8-year cancer event,  mean (SD) | Patients,  #(%) | Probability of 8-year cancer event,  mean (SD) | Patients,  #(%) | Probability of 8-year cancer event,  mean (SD) | Patients,  #(%) | Probability  of 8-year cancer  event,  mean (SD) |
| High (risk score 51.6+) | 132  (10.53) | 0.36  (0.23) | 60  (9.57) | 0.40  (0.28) | 67  (10.69) | 0.38  (0.24) | 291  (9.53) | 0.31  (0.18) |
| Middle (risk score 42.6-51.6) | 964  (76.87) | 0.037  (0.032) | 463  (73.84) | 0.039  (0.033) | 473  (75.44) | 0.038  (0.032) | 2431  (79.63) | 0.040  (0.033) |
| Low (risk score 37.5-42.5) | 158  (12.6) | 0.004  (0.002) | 104  (16.59) | 0.004  (0.001) | 87  (13.88) | 0.005  (0.001) | 331  (10.84) | 0.004  (0.001) |
