# Supplementary material for: Development and validation of a simple risk model to predict major cancers for patients with nonalcoholic fatty liver disease
Source: Cancer Med. 2019 Dec 20;9(3):1254–62. doi: 10.1002/cam4.2777 (PMC6997093; doi:10.1002/cam4.2777)
Supplement: Supplementary file 6 [file CAM4-9-1254-s006.docx]

**eTable 2 The number of patients with each cancer type within each subcohort**

|  | **Total** | **Training** | **Test** | **Evaluation** | **Validation** |
| --- | --- | --- | --- | --- | --- |
| **Liver** | **73** | **26** | **20** | **13** | **14** |
| **Breast** | **65** | **25** | **12** | **14** | **14** |
| **Colon** | **66** | **30** | **9** | **9** | **18** |
| **Prostate** | **36** | **14** | **8** | **8** | **6** |
| **Stomach** | **29** | **17** | **4** | **5** | **3** |
| **Esophagus** | **29** | **10** | **5** | **8** | **6** |
| **Pancreas** | **25** | **5** | **6** | **7** | **7** |
